# Potential factors associated with resilience among older adults in rural China: a multilevel analysis

**DOI:** 10.1186/s12877-023-04575-w

**Published:** 2023-12-12

**Authors:** Yun Qiu, Zhen Cong, Xiaoxuan Wang, Shuzhuo Li

**Affiliations:** 1 School of Public Policy and Administration, Xi’an Jiaotong University, Xi’an, Shaanxi China; 2 School of Public Health, The University of Alabama at Birmingham, Birmingham, AL United States of America; 3 Center for Aging and Health Research, School of Public Policy and Administration, Xi’an Jiaotong University, Xi’an, Shaanxi China

**Keywords:** Rural older adults, Resilience, Residual approach

## Abstract

**Background:**

Resilience is crucial for older adults who experience adversities, but research on the issue in rural China remains limited. This study aims to examine factors associated with resilience among older adults in rural China, as related to different types of resilience, and under different levels of adversity.

**Methods:**

Data were taken from the eight-wave (2001–2021) Longitudinal Study of Older Adults in Anhui Province, China. We used data from the eighth wave (2021) for the outcome variables and lagged predictors (2018) to avoid reverse causal effects. The study sample included individuals 60 years and above, excluding new participants from 2021, those without any adverse events, and any respondents with incomplete analytic data. Resilience was operationalized as residuals of the regressions of life satisfaction (Life Satisfaction Scale) and depressive symptoms (CES-D) on adversity, referred to as Type-1 and Type-2 resilience respectively. These two types of resilience were then treated as the outcome variables in subsequent multilevel regressions, with the predictors focusing on individual, social, and environmental characteristics and resources. This study adheres to STROBE guidelines.

**Results:**

43% of rural older adults exhibited both Type-1 and Type-2 resilience, whereas 18% exhibited only Type-1 resilience and 7% exhibited only Type-2 resilience. Common factors associated with both types of resilience included self-rated health, satisfaction with one’s own financial situation, and the prestigiousness of social networks. Predictors for higher levels of Type-1 resilience included higher levels of financial and emotional support and more options for places of leisure. Predictors for higher levels of Type-2 resilience included greater access to medical care. The prestigiousness of social networks, higher levels of emotional support and instrumental support, access to medical care, and more options of places of leisure were positively associated with resilience in the low-adversity group (first tertile of adversity), only satisfaction with financial situation was positively correlated with the resilience of the middle-adversity group (second tertile), while better self-rated health, satisfaction with financial situation, and financial support yielded greater resilience in the high-adversity group (third tertile).

**Conclusions:**

We examined two types of resilience among older adults in rural China, and found that they have shared and unique associated factors. In addition, the potential factors influencing resilience varied with the level of adversity.

## Background

Population aging has emerged as a prevailing trend in Chinese society in recent years, in conjunction with the transformation of the country’s economy and society [1]. Findings from the Seventh National Population Census (2020) have shown that China has a substantial aging population, with 264 million people aged 60 years and older that represent 18.70% of the country’s population [2]. This represents an increase of 5.44% points compared to the Sixth National Population Census (2010), where those aged 60 years and older comprised 13.26% of the population. Furthermore, the percentage of older adults aged 60 and above is higher in rural areas (23.81%) compared to urban areas (15.82%), with a difference of 7.99% points [3].

### Resilience among residents in rural China

Resilience refers to the capacity to bounce back from adversity or negotiate it more effectively than anticipated, and represents a positive adjustment to challenging circumstances [4–6]. Research on resilience among rural Chinese residents has focused on two key groups: the livelihood resilience of rural households and the resilience of children, especially those left-behind due to parental rural-to-urban migration for employment. Studies on livelihood resilience have delved into how resilience levels affect rural households’ choices of livelihood strategies. These have included pure farming, non-farming occupations, and diversified approaches, which are critical in buffering against economic and environmental adversities [7–9]. Another research area has identified key predictors of livelihood resilience, such as asset endowment and disaster-related factors [10, 11]. Spatio-temporal analysis in the context of livelihood resilience has revealed how livelihood resilience levels fluctuate over time and differ across various rural regions [12–14]. In the area of children’s resilience, studies have highlighted protective factors such as self-efficacy, the frequency of parent-child contact, and a comprehensive understanding of parental labor migration [15–17]. Other studies have addressed the relationship between resilience and mental health issues in children, with an emphasis on serious concerns like suicidal ideation and sleep disturbances [18, 19].

While resilience has been explored across diverse populations within rural China, research specifically targeting the resilience of rural older adults remains scarce. Among the limited studies focusing on this age group, most have concentrated on the impact of resilience on specific outcomes like cognitive functioning and sleep quality [20–22]. Existing research has not fully examined the broader spectrum of challenges faced by rural older adults, which is critical to understanding the full scope of their resilience. Moreover, there is a notable lack of comprehensive analysis regarding the factors that contribute to resilience among rural older adults in China. In-depth knowledge of these resilience factors is crucial, not just for theoretical insight but also for designing effective support programs and interventions that improve the quality of life for rural older adults. Consequently, this paper seeks to bridge this gap by thoroughly exploring the factors associated with resilience among older adults in rural China, with the intention of informing evidence-based strategies to reinforce their resilience.

### Adversity of older adults in rural China

Older adults in rural China are exposed to a variety of adversities. The adversities could include universal stressors and negative life events (i.e., experienced by people of all ages), such as natural disasters, unemployment, and financial problems, as well as age-specific challenges, such as widowhood, chronic illnesses, diminished capacity for self-care, and obstacles to social adaptation [23–25]. In addition, the social networks of older adults in rural China are typically centered on kinship and local connections that feature interactions with neighbors and relatives [26]. This may result in unique adversities, including interpersonal conflicts within social circles and the loss of connections or family members. Furthermore, rural older adults exhibit lower levels of psychological well-being. Evidence from the China Health and Retirement Longitudinal Study (CHARLS) suggests that rural individuals aged 60 years and older (38.3%) are more likely to experience depressive symptoms than their urban counterparts (22.2%) [27]. Finally, resources for older adults in rural areas are relatively limited. For example, rural older adults have significantly lower levels of education and income compared with their urban counterparts [28]. In particular, the most dependable social relationships for rural older adults—their intergenerational relationships—have transformed from a “feedback” model to a model of “intergenerational imbalance” or even “intergenerational exploitation” as a result of diminishing household size, migration of the rural labor force, and weakening of the traditional filial norms [29]. Public services in rural areas are comparatively underdeveloped in terms of the resources available in the environment. This is especially evident in the lower rates of coverage of facilities catering to the needs of older adults in such areas, lower awareness and use of services for older care, and the absence of long-term supportive policies to assist rural residents [30–32]. Moreover, the COVID-19 pandemic has put a strain on the healthcare system that has led to the redistribution of resources and medical personnel to address the disease [33]. Consequently, the scarcity of medical resources in rural areas has been further magnified. The lack of access to necessary medical care may worsen the health-related challenges experienced by rural older adults who rely on long-term medical care services, and may increase their risk of experiencing varying degrees of distress, anxiety, and depressive symptoms [34].

### Factors associated with resilience among older adults

Research on factors related to resilience in older adults has primarily focused on three levels: individual, social, and environmental [35–37]. Individual factors can be classified into subjective and objective. Subjective factors typically reflect older adults’ attitudes toward life [5, 38, 39] while objective factors reflect their objectively measured conditions, such as socioeconomic status [38, 40, 41]. Social factors encompass such resources as social networks and social support for older adults. These social resources can help older adults seek support from others in the face of adversity and trauma to mitigate their isolation, alleviate financial stress, and facilitate the sharing of information and resources [42]. Environmental factors reflect the opportunities for older adults to access public services and engage in social participation, and could include access to public health and geriatric care services as well as surrounding amenities. This is particularly crucial for older adults to maintain autonomy, self-esteem, and meaningfulness [5, 23, 43].

Research on resilience has also shown that different outcomes can be obtained depending on the specific psychological function used to operationalize resilience. This suggests that individuals may exhibit resilience in one domain but not in others. Relying on a single indicator to assess resilience may lead to an inaccurate estimation of the impact of stressors [44]. Cohen et al. found that adolescents display distinct patterns of resilience to post-traumatic stress symptoms (PTSS), depression, violent behavior, and psychological well-being (PWB) following exposure to potentially traumatic events [45]. Hofgaard et al. operationalized resilience based on life satisfaction and internalizing symptoms, and found that these two types of resilience overlap to some extent but also exhibit specificity. Furthermore, the factors associated with these two types of resilience are not entirely identical [46].

Moreover, the factors that promote resilience differ with the level of adversity because of their varying protective effects [6]. Luthar et al. suggested that potential factors can moderate or reduce the impacts of adversity and promote resilience through various protective effects, including the protective stabilizing effect, the protective enhancing effect, the protective reactive effect, and the protective effect [47]. This shows that the effectiveness of the relevant factors may vary depending on the particular circumstances of adversity. In a study on resilience among children, the authors identified self-control as a more significant promoter of resilience in situations involving higher levels of adversity than those involving lower levels of adversity [48]. Identifying the factors associated with different types of resilience, as well as those associated with resilience at varying levels of adversity, can help individuals to decide how and when to mobilize resources, and can provide valuable information to guide precise interventions to improve their resilience.

### The present study

In summary, the existing research provides a foundation for better understanding resilience. However, a majority of findings in the area are based on research by Western scholars on older adults or other age groups in their own countries, and the applicability of their results and insights to older adults in rural China remains to be verified. Consequently, this study addresses three research questions. First, what are the factors associated with resilience among older adults in rural China? Second, do disparities exist in the manifestation of resilience among older adults when different psychological functions are used to operationalize the concept, and are there differences in the potential factors influencing different types of resilience? Third, are there differences in factors related to resilience among rural older adults under different levels of adversity?

## Methods

### Setting

The data used in this study were taken from the Longitudinal Study of Older Adults in Anhui province, China. Anhui is a traditional agricultural region located in eastern China. As of the end of 2010, the percentages of people in the province aged 60 years and older, and 65 years and older to the total resident population were 15.01% and 10.23%, respectively, indicating the emergence of an aging society [49, 50]. The high level of rural-to-urban migration among Anhui’s working-age adults was indicative of broader trends across rural China and has led to significant shifts in family structure and intergenerational interactions [51]. We used a multistage stratified sampling approach to capture a representative cross-section of Anhui’s diverse rural demographics. Initially, 12 townships were randomly chosen from Anhui’s 126 rural townships. Subsequently, 6 villages were randomly picked from each township. Finally, two age-based groups of rural residents aged 60 and older were compiled for sampling: one group aged 60–74 and another 75 years and older. The baseline sample was recruited in 2001 by using a stratified, multistage methodology, and follow-up surveys were conducted in 2003, 2006, 2009, 2012, 2015, 2018, and 2021. Four replenishment subsamples were added in 2009, 2015, 2018, and 2021.

### Participants

The analysis included individuals aged 60 years and above. We constructed a two-wave longitudinal subset from the 2018 and 2021 data waves. This lagged design, with resilience variables measured in 2021 and predictors measured in 2018, was utilized to mitigate reverse causality. Initially, we excluded new participants from the 2021 wave who lacked corresponding data from 2018. Furthermore, we removed the responses of older adults who had not experienced any adversity, aligning with our operational definition of resilience which presupposes the existence of challenging circumstances. Lastly, we excluded respondents with incomplete data for the analytical variables.

### Measures

#### Operationalization of resilience

The resilience of older adults in rural China was operationalized through the “residual approach” [52]. It describes the idea of an increase in the average expected negative outcomes as the level of adversity increases, but the average may obscure individual variations at the same level of adversity. In other words, different individuals may deviate from the expected outcome, i.e., they may exhibit better or worse outcomes than expected at the same level of adversity. This deviation was defined as resilience [53]. Following this idea, resilience can be operationalized as the residual value of the regression of the psychological function on adversity. If the value of the residual was positive, the corresponding individuals were considered to be “resilient,” indicating better outcomes than those predicted by the regression model [54].

#### Adversity

Adversity was measured as the sum of two components, negative life events within the past year and lifetime stressors, by using data from 2021. First, negative life events were identified by asking the older adult about events that they had experienced within the past year. The checklist considered previous research in the context of rural China [55–57], including (1) serious diseases, (2) natural disasters, (3) deaths of relatives or friends, (4) conflicts with relatives or friends, (5) accidents, (6) retirement, and (7) other important negative life events. Second, stressors for older adults during their lifetime have been investigated in research on resilience [58–61], including (8) widowhood, (9) loss of a child, (10) chronic illness, (11) limitations in ADL (basic activities of daily living), (12) limitations in IADL (instrumental activities of daily living), (13) ADL-related limitations of the spouse, (14) IADL-related limitations of the spouse, and (15) marital conflicts involving adults children (1 = divorced or separated due to marital problems/widowed/never married and 0 = married and spouse was alive). Adversity was calculated based on the total scores of the respondents on the above life events and stressors, with a range of values of 0–15. Higher scores signified elevated levels of adversity.

#### Different psychological functions

To distinguish between the resilience of different psychological functions, we referred to the residuals of life satisfaction as Type-1 resilience and those associated with depressive symptoms as Type-2 resilience [46]. As the residuals of the regressions on adversity and depressive symptoms were negative for resilience, Type-2 resilience was obtained by reversing the residuals.

Life satisfaction reflects the cognitive evaluation of individuals’ current quality of life and depressive symptoms assess their short-term emotional distress. Life satisfaction was evaluated based on eight items adapted from the Satisfaction With Life Scale [62], which asked respondents whether they agreed or disagreed with statements related to their current lives (better life than most people, satisfied with life, interesting life, best years of life, willing to change past life, tedious, tiring, and life meets expectations). The responses were coded as 1 (agree) or 0 (disagree) for each item. The coding of items representing discontentment was reversed (i.e., willing to change past life, tedious, tiring) and summed to create a scale of life satisfaction ranging from 0 (least satisfied) to 8 (most satisfied).

Depressive symptoms were measured by an adapted nine-item version of the Center for Epidemiologic Studies–Depression Scale [63, 64]. It consisted of three items of positive affect (good mood, having a good life, and pleasantness), two items of negative affect (loneliness and upset), two items of the marginalization of emotions (useless, and having nothing to do), and two items of somatic symptoms (poor appetite and insomnia). The participants were asked to rate the frequency of each depressive symptom in the past week by using a coding system of 0 (rarely or never), 1 (some of the time), or 2 (most of the time). Following the reverse coding of the three items reflecting positive affect, the nine items were summed to create a scale of depressive symptoms, with scores ranging from 0 (least depressive symptoms) to 18 (most depressive symptoms).

### Potential factors associated with resilience

#### Individual factors

The socioeconomic status of the respondents was evaluated by their income and education. Income was measured by summing the earnings of both the respondents and their spouses from work, pension, and retirement benefits received within the past 12 months. Their total income was then natural log-transformed to reduce the skewness and discreteness of the data. Education was coded as 1 = some education and 0 = no formal education.

Satisfaction with financial situation was measured by one global item as well—“Generally speaking, are you satisfied with your present financial situation?“—on a four-point scale (1 = very unsatisfied, 2 = unsatisfied, 3 = satisfied, and 4 = very satisfied).

Self-rated health was measured by one global item—“What do you think about your health status?“—on a four-point scale (1 = not so good, 2 = just so-so, 3 = good, and 4 = very good). The validity of this single-item measure has been demonstrated for older Chinese adults [65–67].

The respondents’ attitudes toward aging were measured by an adapted 12-item version of the Attitudes to Ageing Questionnaire [68]. It consisted of eight items of positive attitudes (importance of exercise, not feeling old, identity is not defined by age, engaging in desired activities even with health problems, coping with life better as they get older, wisdom comes with age, sharing experiences, giving a good example) and four items of negative attitudes (loneliness, depressing time of life, difficult to talk about feelings, not involved in society). The responses were assessed on a five-point scale, ranging from 1 (strongly disagree) to 5 (strongly agree). After the reverse coding of the items reflecting negative attitudes, the 12 items were summed to generate a scale of attitudes toward aging ranging from 12 to 60, with higher scores indicating more positive attitudes towards aging. The attitudes toward aging were categorized into tertiles, with the first tertile representing the most negative category of attitudes and the third tertile representing the most positive category.

#### Social support

Social support encompassed both the characteristics of the respondents’ social networks and the intergenerational support for them. The characteristics of the social network included its size and prestigiousness. The size of the respondents’ social networks was assessed by using the Friends subscale from Lubben’s Social Network Scale [69]. It contained three questions: (a) “How many of your friends do you see or hear from at least once a month?” (b) “How many friends do you feel at ease with such that you can talk to them about private matters?” and (c) “How many friends do you feel close to such that you could call on them for help?” For each question, the responses were coded as 0 = 0, 1 = 1, 2 = 2, 3 = 3 or 4, 4 = 5–8, and 5 = 9 and above. Following Lubben’s recommendations, the responses were summed to indicate the size of the social network, ranging from 0 (minimal size) to 15 (maximal size).

The prestigiousness of social networks was measured by asking them: “How many relatives do you have in village cadres, township cadres, and among other national public officials?” Their responses were dichotomized and coded as follows: 0 = none, and 1 = at least one.

Intergenerational support for the respondents was assessed based on the emotional, financial, and instrumental support that they received from their adult children. Emotional support was assessed by using three questions: (a) “Generally, do you feel close with this child?” (b) “Generally, do you feel that you are on good terms with this child” and (c) “How much do you feel that this child would be willing to listen when you need to talk about your worries and problems?” The responses were coded as follows: 0 = not at all close/no at all well/not at all, 1 = close to some degree/just so-so/sometimes, and 2 = very close/very well/very much, respectively. An additive scale ranging from 0 to 6 was computed for each child of each older adult respondent. We took the tertile of the sum of emotional support received by each parent from all their children to calculate this construct, and it was coded as follows: 1 = low emotional support, 2 = moderate emotional support, and 3 = high emotional support.

The financial support available to older Chinese adults was assessed based on the total amount that each respondent had received from each of their children within the past 12 months. We took the tertile of the total amount received by each parent from all their children to calculate this construct, which was coded as follows: 1 = low financial support, 2 = moderate financial support, and 3 = high financial support.

The instrumental support provided to the respondents was assessed based on whether they had received support from their adult children, relatives, or neighbors within the past 12 months in the following two domains: (a) household tasks, such as cleaning the house and washing clothes, and (b) personal care tasks, such as bathing and dressing. If the respondent had received any help during the past 12 months, this was coded as 1, and was otherwise 0.

#### Environmental factors

Environmental factors included the variables used to characterize the villages/ village committees.

Access to medical care for older Chinese adults was determined by the presence of a hospital, infirmary, health room, or clinic within the village/village committee. If any of these facilities was present, the code for it was assigned the value 1, and was otherwise assigned 0.

Access to geriatric care services was determined by the presence of a daycare center for older adults or a retirement home within the given village/village committee. If any of these facilities was present, the code was set to 1, and was otherwise set to 0.

Options of places of leisure available to the respondents were assessed by the number of leisure facilities, including gyms, recreation centers for older adults, libraries, parks, chess and card game rooms, and public areas for exercise. The scores ranged from 0 to 6, with higher scores indicating more options of places of leisure.

#### Covariates

The covariates included age and gender (0 = male, 1 = female). Age was categorized into three groups for analysis: ‘60–69 years old’ (coded as 0), ‘70–79 years old’ (coded as 1), and ‘80 years old and above’ (coded as 2).

### Statistical analysis

First, descriptive statistics were used to describe adversity, psychological functions, and the factors that may influence resilience. Continuous variables were summarized by their mean values (standard deviation, SD) and categorical variables were displayed as frequencies (percentages, %).

Second, based on the residual approach, we used life satisfaction and depressive symptoms as separate outcome variables in univariate linear regression, with adversity as the sole independent predictor variable, to operationalize resilience. We defined the residuals from the regression of life satisfaction on adversity as Type-1 resilience and those from the regression of depressive symptoms on adversity as Type-2 resilience. Subsequent descriptive analysis was conducted to examine the characteristics of Type-1 and Type-2 resilience.

Third, to determine the appropriateness of multilevel modelling, we calculated the Intraclass Correlation Coefficients (ICCs) using the one-way random effects model before introducing any independent variables [70]. The calculation aimed to discern the variance in resilience ratings attributable to differences between villages, as opposed to within-village variability. The ICC was computed using the formula: $\text{I}\text{C}\text{C} (1, 1) = {\sigma }_{b}^{2}/\left({\sigma }_{b}^{2}+{\sigma }_{w}^{2}\right)$, where ${\sigma }_{b}^{2}$ represented the between-village variance component, and ${\sigma }_{w}^{2}$ represented the within-village variance component [71]. A threshold ICC value of 0.059, as recommended by the cited guidelines, was used to determine the need for multilevel modelling [72]. Having established the necessity for multilevel modelling, Type-1 resilience (Model 1) and Type-2 resilience (Model 5) were used as the outcome variables in the multilevel regression analysis of the whole sample, with the proposed potential factors entered as predictor variables to identify the associated factors. To avoid reverse causality, the predictor variables were measured using data from 2018.

Fourth, to determine if the effects of potential factors on resilience varied with adversity levels among older adults, we utilized a tertile approach to categorize adversity scores. Specifically, adversity scores were divided into tertiles, forming three distinct groups representing varying levels of adversity: the lowest third of participants by score were categorized into the low-adversity group (Model 2 and Model 6), the middle third into the middle-adversity group (Model 3 and Model 7), and the highest third into the high-adversity group (Model 4 and Model 8). This method allowed for a stratified analysis to assess the influence of resilience factors within each adversity context.

We adopted a *p*-value threshold of less than 0.05 to determine statistical significance across all analyses. All analyses were carried out in STATA 16.0.

## Results

### Participants

The 2021 survey involved 1,560 respondents and contained 1,550 valid responses. From these, we excluded 570 individuals who were newly added to the survey in 2021. Additionally, 84 older adults who had not encountered adverse events were removed from the study cohort. A further 12 respondents were excluded due to missing data on any essential analytic variables. Following these exclusions, the final analytical sample consisted of 884 older adults.

### Descriptive statistics

Table 1 presents the characteristics of older adults in rural China, including those of the whole sample and subgroups based on the levels of adversity. Older adults in rural areas aged 60–69, 70–79, and 80 years and above constituted 54.30%, 31.45%, and 14.25%, respectively, of the whole sample. Females constituted 47.40%. Based on the adversity, the older adults were categorized into a low-adversity group (first tertile), a middle-adversity group (second tertile), and a high-adversity group (third tertile) with respective adversity scores of 2.73, 3.00, and 4.54, respectively.

**Table 1:** Characteristics of the participants by the adversity groups

	Whole sample (N = 884)		Low adversity (N = 428)		Middle adversity (N = 205)		High adversity (N = 251)	
	Mean/N	SD/%	Mean/N	SD/%	Mean/N	SD/%	Mean/N	SD/%
**Adversity (2021)**	2.73	1.40	1.53	0.50	3.00	0.00	4.54	0.85
**Psychological function (2021)**								
Life satisfaction	5.31	2.21	5.75	1.94	5.29	2.22	4.58	2.43
Depressive symptoms	5.50	3.71	4.10	3.03	5.82	3.30	7.64	3.98
**Potential factors (2018)**								
**Individual factors**								
Income	8.45	1.60	8.68	1.67	8.25	1.58	8.19	1.41
Education (some education)	383	43.33	228	53.27	70	34.15	85	33.86
Satisfaction with financial situation	3.02	0.65	3.06	0.64	3.06	0.65	2.92	0.65
Self-rated health	2.46	0.99	2.68	0.99	2.42	0.97	2.12	0.92
Attitudes toward aging								
1st tertile (negative)	329	37.22	148	34.58	77	37.56	104	41.43
2nd tertile	289	32.69	137	32.01	75	37.07	76	30.28
3rd tertile (positive)	266	30.09	143	33.41	52	25.37	71	28.29
**Social support**								
Size of social network	5.37	3.50	5.47	3.54	5.06	3.35	5.44	3.56
Prestigiousness of social networks (high)	246	27.83	114	26.64	55	26.83	77	30.68
Emotional support								
1st tertile (lowest)	405	45.81	222	51.87	82	40.00	101	40.24
2nd tertile	242	27.38	126	29.44	63	30.73	53	21.12
3rd tertile (highest)	237	26.81	80	18.69	60	29.27	97	38.65
Financial support								
1st tertile (lowest)	329	37.22	158	36.92	78	38.05	93	37.05
2nd tertile	262	29.64	123	28.74	63	30.73	76	30.28
3rd tertile (highest)	293	33.14	147	34.35	64	31.22	82	32.67
Instrumental support (yes)	273	30.88	119	27.80	64	31.22	90	35.86
**Environmental factors**								
Access to medical care (at least one)	750	84.84	360	84.11	175	85.37	215	85.66
Access to geriatric care services (at least one)	104	11.76	57	13.32	25	12.20	22	8.76
Options of places of leisure	2.56	1.70	2.50	1.72	2.46	1.65	2.76	1.69
**Control variable**								
Age								
60–69 years old	480	54.30	296	69.16	89	43.41	95	37.85
70–79 years old	278	31.45	111	25.93	70	34.15	97	38.65
80 years old and above	126	14.25	21	4.91	46	22.44	59	23.51
Gender (female)	419	47.40	152	35.51	112	54.63	155	61.75

SD: Standard Deviation

### Internal reliability

We assessed the internal reliability of our measurement tools and report their Cronbach’s alpha values as follows: The Satisfaction with Life Scale (2021) had a Cronbach’s alpha of 0.79, indicating good reliability. The nine-item version of the Center for Epidemiologic Studies–Depression Scale (2021) also showed good reliability with a Cronbach’s alpha of 0.79. The Attitudes to Ageing Questionnaire (2018) had a Cronbach’s alpha of 0.72, while the Friends Subscale (2018) and the emotional support items (2018) recorded Cronbach’s alpha values of 0.84 and 0.81, respectively, both signifying strong reliability.

#### Resilience analysis

Table 2 displays the outcomes of the residual approach for Type-1 and Type-2 resilience.

**Table 2:** Residual approach for Type-1 and Type-2 resilience

**Type-1 resilience**			
Life satisfaction	Coef.	95% Confidence Interval	*p*-value
Adversity	-0.38	[-0.48, -0.28]	< 0.001
Observations	884		
**Type-2 resilience**			
Depressive symptoms	Coef.	95% Confidence Interval	*p*-value
Adversity	1.10	[0.95, 1.26]	< 0.001
Observations	884		

Having obtained the scores for both types of resilience, we then proceeded to conduct a descriptive analysis to further explore the characteristics of Type-1 and Type-2 resilience. Only 43% of older adults exhibited resilience (i.e., with residual values greater than 0) of both types, where this was associated with higher life satisfaction and lower depressive symptoms than predicted. The percentage of older adults who demonstrated a lack of resilience (i.e., with residual values smaller than 0) of both types was 32%, and this was associated with lower life satisfaction and greater depressive symptoms than predicted. Only 17% of adults exhibited Type-1 resilience (which was correlated with higher life satisfaction but higher depressive symptoms than predicted) while 8% exhibited only Type-2 resilience (which was correlated with lower life satisfaction but lower depressive symptoms than predicted). Table 3 shows the ranges of Type-1 and Type-2 resilience in the whole sample as well as in each adversity-related group.

**Table 3:** Characteristics of Type-1 and Type-2 resilience by the adversity groups

	Whole sample (N = 884)		Low adversity (N = 428)		Middle adversity (N = 205)		High adversity (N = 251)	
	Min	Max	Min	Max	Min	Max	Min	Max
**Type-1 resilience**	-5.97	3.93	-5.97	2.41	-5.21	2.79	-4.83	3.93
**Type-2 resilience**	-10.09	9.11	-7.40	4.70	-8.20	5.80	-10.09	9.12

### Multilevel regression analyses

Before any independent variable was added, the ICCs for resilience among older adults in different villages/village committees were 0.19 (Type-1 resilience) and 0.15 (Type-2 resilience), indicating that 19% and 15% of the overall variations in resilience had occurred due to variations between villages/village committees.

Table 4 displays the results of multilevel regression analyses to examine the associations between Type-1 resilience and the potential factors influencing it, with Model 1 representing the whole sample and Models 2–4 representing each adversity-related group. Self-rated health, satisfaction with financial situation, and prestigiousness of social networks, emotional support, financial support, and options of places of leisure had significant positive effects on resilience in the whole sample set (Model 1). The prestigiousness of social networks, emotional support, and options of places for leisure had significant positive effects on respondents in the low-adversity group (Model 2). Satisfaction with financial situation had significant positive effects on the middle-adversity group (Model 3), while self-rated health and satisfaction with financial situation had significant positive effects on the high-adversity group (Model 4). Differences in factors that were significant across Models 1–4 suggested that the factors promoting Type-1 resilience varied with the level of adversity. Notably, access to geriatric care services had a significant negative effect on Type-1 resilience in Models 1–4.

**Table 4:** Multilevel linear regression analysis of Potential factors for Type-1 resilience

Potential factors	Model 1 (Whole sample)		Model 2 (Low adversity)		Model 3 (Middle adversity)		Model 4 (High adversity)	
	Coef.	*p*-value	Coef.	*p*-value	Coef.	*p*-value	Coef.	*p*-value
**Fixed effects**								
**Individual factors**								
Income	0.02	0.727	0.00	0.972	0.10	0.340	-0.03	0.797
Education (ref = no)								
Some education	0.26	0.093	0.21	0.274	0.33	0.350	0.23	0.476
Satisfaction with financial situation	0.43	**< 0.001**	0.15	0.324	0.73	**0.002**	0.70	**0.004**
Self-rated health	0.23	**0.002**	0.12	0.195	0.25	0.122	0.46	**0.007**
Aging attitude (ref = 1st tertile)								
2nd tertile	-0.04	0.803	0.12	0.577	-0.20	0.556	-0.36	0.293
3rd tertile (highest)	0.03	0.884	0.27	0.247	-0.09	0.819	-0.47	0.208
**Social support**								
Size of social network	-0.03	0.127	-0.01	0.650	-0.02	0.711	-0.08	0.080
Prestigiousness of social networks (ref = low)								
High	0.33	**0.029**	0.46	**0.022**	0.02	0.954	0.55	0.090
Emotional support (ref = 1st tertile)								
2nd tertile	0.35	**0.033**	0.54	**0.009**	0.24	0.470	0.17	0.668
3rd tertile (highest)	0.31	0.096	0.51	**0.049**	0.40	0.312	-0.18	0.629
Financial support (ref = 1st tertile)								
2nd tertile	0.05	0.756	0.14	0.516	0.30	0.390	-0.43	0.227
3rd tertile (highest)	0.41	**0.018**	0.25	0.266	0.32	0.407	0.64	0.075
Instrumental support (ref = no)								
Yes	-0.09	0.562	0.14	0.491	0.16	0.622	-0.19	0.536
**Environmental factors**								
Access to medical care (ref = none)								
At least one	0.24	0.450	0.38	0.244	-0.11	0.839	0.16	0.733
Access to geriatric care services (ref = none)								
At least one	-1.44	**< 0.001**	-1.26	**0.001**	-1.75	**0.011**	-1.61	**0.005**
Options of places of leisure	0.17	**0.012**	0.16	**0.026**	0.21	0.094	0.18	0.068
**Control variable**								
Age (ref = 60–69 years old)								
70–79 years old	-0.21	0.166	-0.73	**< 0.001**	0.28	0.405	-0.03	0.939
80 years old and above	0.13	0.548	-0.20	0.637	0.15	0.724	0.25	0.556
Gender (ref = male)								
Female	0.02	0.886	0.02	0.932	-0.34	0.313	0.25	0.430
**Intercept**	-2.76	**< 0.001**	-1.90	**0.011**	-4.08	**0.001**	-3.10	**0.010**
**Random effects**								
Environmental factors variance	0.61		0.48		1.11		0.46	
**Observations**	884		428		205		251	
**Model fit**								
-2LL	3674.72		1690.93		860.33		1099.25	
AIC	3718.72		1734.93		904.33		1143.25	
BIC	3823.98		1824.23		977.44		1220.81	

-2LL: -2 Log Likelihood. AIC: Akaike’s Information Criterion. BIC: Schwarz’s Bayesian Criterion

Bold face indicates statistical significance (*p* < 0.05)

Table 5 presents the results of multilevel regression analyses of the associations between Type-2 resilience and the factors potentially influencing it, with Models 5–8 representing the whole sample and subgroups based on the levels of adversity. Self-rated health, satisfaction with financial situation, prestigiousness of social networks, and access to medical care had significant positive effects on the whole sample set (Model 5). Similarly to Type-1 resilience, factors promoting Type-2 resilience varied across levels of adversity. The prestigiousness of social networks, instrumental support, and access to medical care had significant effects on the low-adversity group (Model 6). No factor was found to significantly promote resilience in the middle-adversity group (Model 7), while self-rated health, satisfaction with financial situation, and financial support had significant effects on the high-adversity group (Model 8).

**Table 5:** Multilevel linear regression analysis of potential factors for Type-2 resilience

Potential factors	Model 5 (Whole sample)		Model 6 (Low adversity)		Model 7 (Middle adversity)		Model 8 (High adversity)	
	Coef.	*p*-value	Coef.	*p*-value	Coef.	*p*-value	Coef.	*p*-value
**Fixed effects**								
**Individual factors**								
Income	0.06	0.432	0.02	0.811	0.07	0.648	0.01	0.942
Education (ref = no)								
Some education	0.47	0.057	0.32	0.271	0.97	0.091	0.51	0.337
Satisfaction with financial situation	0.51	**0.005**	0.10	0.642	0.57	0.134	0.96	**0.015**
Self-rated health	0.34	**0.004**	0.13	0.356	0.42	0.100	0.77	**0.005**
Aging attitude (ref = 1st tertile)								
2nd tertile	0.23	0.390	0.63	0.054	-0.33	0.541	-0.21	0.703
3rd tertile (highest)	0.15	0.612	0.31	0.378	-0.06	0.929	-0.03	0.967
**Social support**								
Size of social network	-0.02	0.620	0.04	0.344	0.02	0.809	-0.13	0.082
Prestigiousness of social networks (ref = low)								
High	0.61	**0.013**	0.79	**0.008**	0.54	0.294	0.41	0.436
Emotional support (ref = 1st tertile)								
2nd tertile	-0.03	0.896	0.31	0.309	0.03	0.954	-0.15	0.816
3rd tertile (highest)	0.31	0.304	0.32	0.401	0.75	0.234	-0.12	0.851
Financial support (ref = 1st tertile)								
2nd tertile	-0.25	0.356	-0.25	0.444	-0.16	0.776	-0.54	0.352
3rd tertile (highest)	0.43	0.125	0.06	0.849	-0.55	0.365	1.70	**0.004**
Instrumental support (ref = no)								
Yes	0.12	0.613	0.60	**0.046**	0.08	0.876	0.05	0.916
**Environmental factors**								
Access to medical care (ref = none)								
At least one	1.30	**0.007**	1.74	**0.004**	0.44	0.556	1.33	0.091
Access to geriatric care services (ref = none)								
At least one	-0.97	0.081	-1.08	0.115	-0.74	0.435	-1.05	0.281
Options of places of leisure	0.12	0.275	0.11	0.390	0.08	0.639	0.07	0.692
**Control variable**								
Age (ref = 60–69 years old)								
70–79 years old	-0.50	**0.044**	-0.96	**0.001**	-0.04	0.948	-0.78	0.164
80 years old and above	-0.47	0.180	-1.28	**0.041**	-0.19	0.773	-0.86	0.220
Gender (ref = male)								
Female	-0.28	0.240	-0.52	0.072	-0.02	0.968	-0.21	0.689
**Intercept**	-4.38	**< 0.001**	-3.04	**0.009**	-4.26	**0.029**	-5.18	**0.009**
**Random effects**								
Environmental factors variance	1.46		2.17		1.18		1.92	
**Observations**	884		428		205		251	
**Model fit**								
-2LL	4512.72		2038.49		1047.81		1347.31	
AIC	4556.72		2082.49		1091.81		1391.31	
BIC	4661.98		2171.79		1164.92		1468.87	

-2LL: -2 Log Likelihood. AIC: Akaike’s Information Criterion. BIC: Schwarz’s Bayesian Criterion

Bold face indicates statistical significance (*p* < 0.05)

## Discussion

In this study, we operationalized the concept of resilience as residuals from the linear regressions of the adversity on life satisfaction (i.e., Type-1 resilience) and depressive symptoms (i.e., Type-2 resilience). Factors influencing these two types of resilience were analyzed by using models of multilevel regression for older adults in rural China. Furthermore, disparities in the factors associated with resilience at different levels of adversity were identified. The results lead to the following three conclusions centering on the proposed research questions.

First, factors associated with resilience among older adults in rural China could be identified across all three levels, i.e., individual, social, and environmental, which is consistent with the results of past research and provides further support for a comprehensive framework and approach for resilience research [35–37]. Among individual factors, self-rated health and satisfaction with financial situation had positive effects on both types of resilience, and the results verified their importance in promoting resilience especially among older adults with higher level of community-related adversity. Previous studies have shown that self-rated health not only reflects the objective physical condition of older adults, but also their subjective attitudes toward their own condition [73, 74]. For example, some older adults may perceive the decline in their physical condition as a normal age-related change compared with their peers [39]. Satisfaction with financial situation reflects the attitudes of older individuals toward their actual income and the level of security they feel regarding their financial situation later in life [75]. The actual income of older adults did not influence their resilience, indicating that subjective attitudes toward their financial status may more accurately reflect the contextual living conditions of older adults than objective measures of income. The latter is limited in measuring their actual living conditions, e.g., because of their different living expenses. In contrast, education, another factor reflecting their socioeconomic status, did not have a significant relationship with resilience among older adults in rural China according to this study. There are two possible explanations for this: On the one hand, education typically represents an individual’s capacity to acquire resources; however, older adults in China at present, particularly the oldest, were raised before the People’s Republic of China was established in 1949, and spent their adulthood during the period of a planned economy, or collectivism [76]. Coupled with the limited resources in rural areas, this might have hindered local residents from capitalizing on their advantage in education for resource acquisition. On the other hand, an individual’s subjective sense of well-being depends on the fulfillment of their expectations, and education elevates people’s expectations for their own future development. This can lead to difficulties in adapting to adversity in later life. Another possible explanation lies in the homogeneity of educational levels among the respondents. Among the 884 respondents in the sample, 383 (43%) had received formal schooling, of which 301 (78% of those educated) had only received primary education. This indicates a predominance of lower educational qualifications in the sample. The limited variation in education levels might partially explain why education does not appear to have a significant effect on resilience among the respondents.

Among social factors, the prestigiousness of social networks had positive effects on both types of resilience. Social networks that include people with prestigious status could provide older adults in rural China with access to resources, thus enabling them to adopt various adaptive strategies to cope with adverse events in life and demonstrate higher levels of resilience [77, 78]. Financial support and emotional support had a positive association with Type-1 resilience, reflecting the importance of intergenerational support for resilience among older people in rural China. Due to Chinese culture norms of filial piety, older adults in rural areas still have high expectations of intergenerational support from their adult children [79]. First, older adults in rural areas frequently encounter restricted individual and environmental resources, and financial support from their adult children can fulfill their material needs. According to the Seventh National Population Census, in 2020, family support was the primary source of livelihood for adults aged 60 years and older in rural areas, and the ratio of familial support to their overall livelihood gradually increased with age [80]. In addition, emotional support can mitigate feelings of loneliness, helplessness, and anxiety caused by aging. Older adults tend to address emotional concerns as an effective strategy for resilience, particularly when the stress-inducing environment cannot be changed [58, 81]. Emotional support is particularly significant for older adults in China as it serves as a vital expression of filial piety. Financial and instrumental support provided to older adults must be grounded in respect and love [82].

Among environmental factors, access to medical care had a positive effect on resilience that was manifested in two ways. Access to medical care was closely related to resilience among older adults in the context of daily disease management, prevention of chronic diseases, delaying functional decline, provision of long-term care, and ensuring access to basic emergency care [36, 43, 83]. In addition, when medical care services within the residential area could meet the needs of geriatric care, older adults could continue to maintain interactions within their social networks, thereby preserving social resources to cope with adversity [84]. The options of places of leisure had a positive effect on resilience as well. More options of places of leisure can provide various avenues for social participation for older adults in rural areas, thus facilitating the establishment of interpersonal relationships and social activities that are crucial for building resilience.

The negative effect of access to geriatric care services on resilience among older adults in rural China was noteworthy. There are two possible reasons for it. First, older adults are often hesitant to leave familiar environments, and moving into a geriatric care institution may disrupt their social networks and necessitate adjustments to their living environment [85–87]. Second, the norms of filial piety in Chinese culture are the basis of the expectations of support among older adults from their children. In particular in rural areas, where filial piety is particularly strong, older people who have children and move to care institutions may be considered “unfortunate” or “abandoned by their families.” [88, 89] In this context, village-based care institutions may not be able to compensate for the loss of social interactions, and could also cause stigma and certain psychological problems that ultimately lead to lower levels of resilience.

**Fig. 1:**
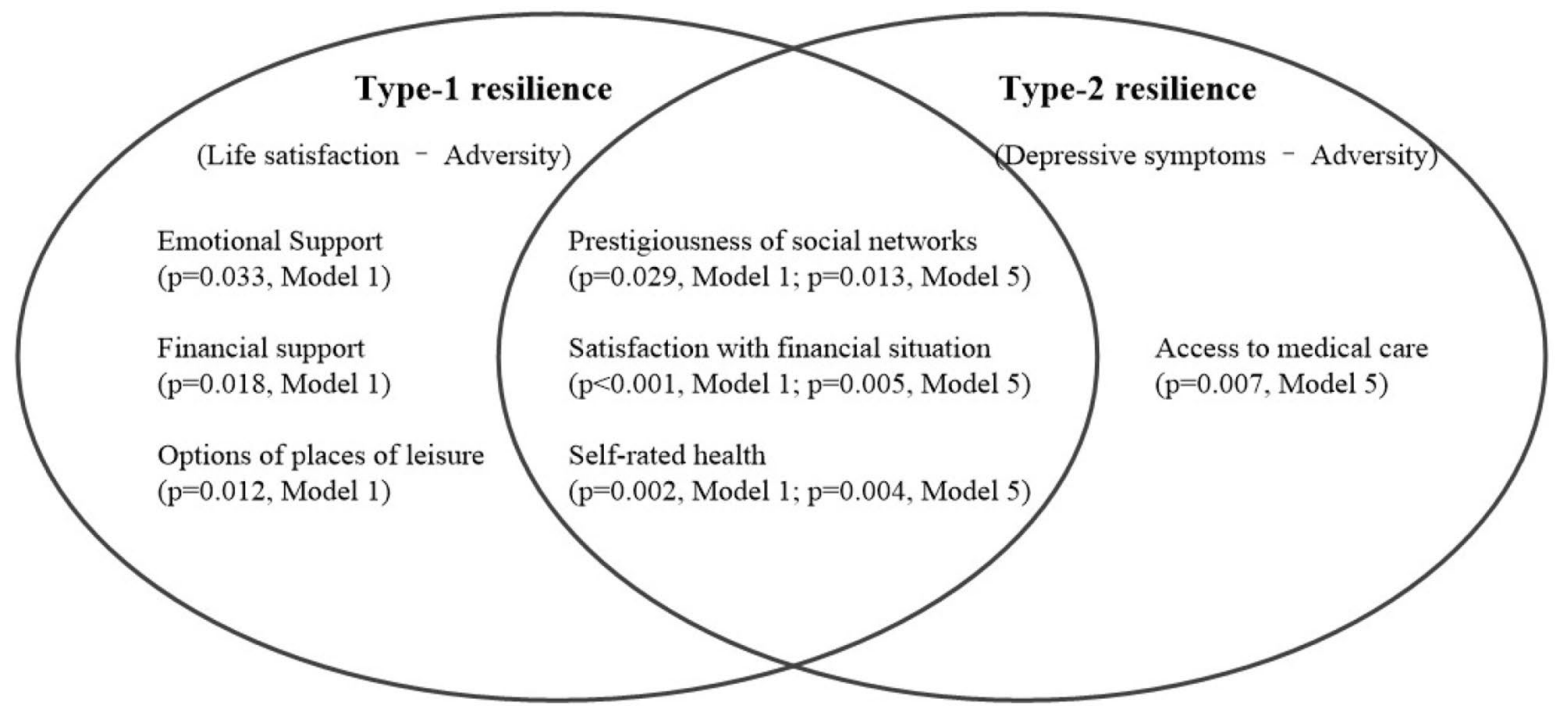
Significant factors of Type-1 and Type-2 resilience This Venn diagram illustrates the statistically significant factors (*p* < 0.05) that contribute to Type-1 and Type-2 resilience among older adults in rural China. Type-1 resilience is operationalized as the residuals of life satisfaction on adversity, whereas Type-2 resilience refers to the residuals of depressive symptoms on adversity. The left circle presents factors that predict Type-1 resilience, the right circle those for Type-2 resilience, and the overlapping area shows predictors common to both resilience types. Notably, ‘Access to geriatric care services’ is not depicted due to its statistically significant negative impact on resilience Model 1: Multilevel linear regression of Type-1 resilience of the whole sample Model 5: Multilevel linear regression of Type-2 resilience of the whole sample

Second, our findings suggest that it is important to examine different types of resilience among rural older adults in China. The coefficient of correlation between Type-1 resilience and Type-2 resilience was 0.61, implying overlaps between these two constructs as well as the uniqueness of each. Figure 1 illustrates the shared and unique predictors of the two types of resilience. Predictors shared by both types of resilience in the whole sample set included self-rated health, satisfaction with financial situation, and prestigiousness of social networks. This indicates the importance of attitudes toward life in mitigating external stress and thereby sustaining life satisfaction while avoiding depressive symptoms. Moreover, the social network of rural older adults primarily originates from kinship, and having relatives who serve in village cadres or other national public offices can help facilitate their access to information and resources [26, 77, 78]. Predictors specific to Type-1 resilience were financial support, emotional support, and options of places of leisure, whereas access to medical care was specific to Type-2 resilience.

The above result can be associated with the way to define the two types of resilience. The psychological functions used to operationalize resilience—namely, life satisfaction and depressive symptoms—reflect the positive and negative aspects of psychological functioning, respectively. They are not opposite constructs, and are not two poles of a continuum [90, 91]. The dual-factor model of mental health proposes that both positive subjective well-being and negative pathological indicators are inseparable components of an individual’s psychological function [92].

Life satisfaction is defined as a stable, long-term cognitive judgment of one’s quality of life, shaped by personal values and criteria [93]. This reflects an enduring perception of well-being. Conversely, depressive symptoms are defined as fluctuating emotional states, typically transient, and characterized by feelings such as sadness, hopelessness, and a lack of interest or pleasure in activities [63, 94]. These symptoms are indicative of short-term emotional reactions rather than prolonged psychological conditions. Thus, in the context of this study, resilience implied that despite adversities, an individual’s assessment of their quality of life was better than expected (Type-1 resilience), and/or that their emotional distress was lower than anticipated (Type-2 resilience). Different conceptual meanings of these two types of resilience ultimately manifested as variations in factors associated with an individual’s resilience across those two different psychological functions. Some findings can be further examined for future theoretical development in this vein. Particularly, the results showed that intergenerational support provided to older adults by their adult children enhanced only Type-1 resilience. This suggests that the support provided by their children during challenging times has a notable positive impact on the quality of life of older adults, but does not have a significant effect on mitigating their negative emotions. Access to medical care was found to be associated only with Type-2 resilience, possibly because physical health-related issues are a primary source of negative emotions in later life. Medical institutions within the village/village committee can ensure timely access to medical care for older adults while reducing the anxiety and fear associated with their inability to find timely medical attention.

**Fig. 2:**
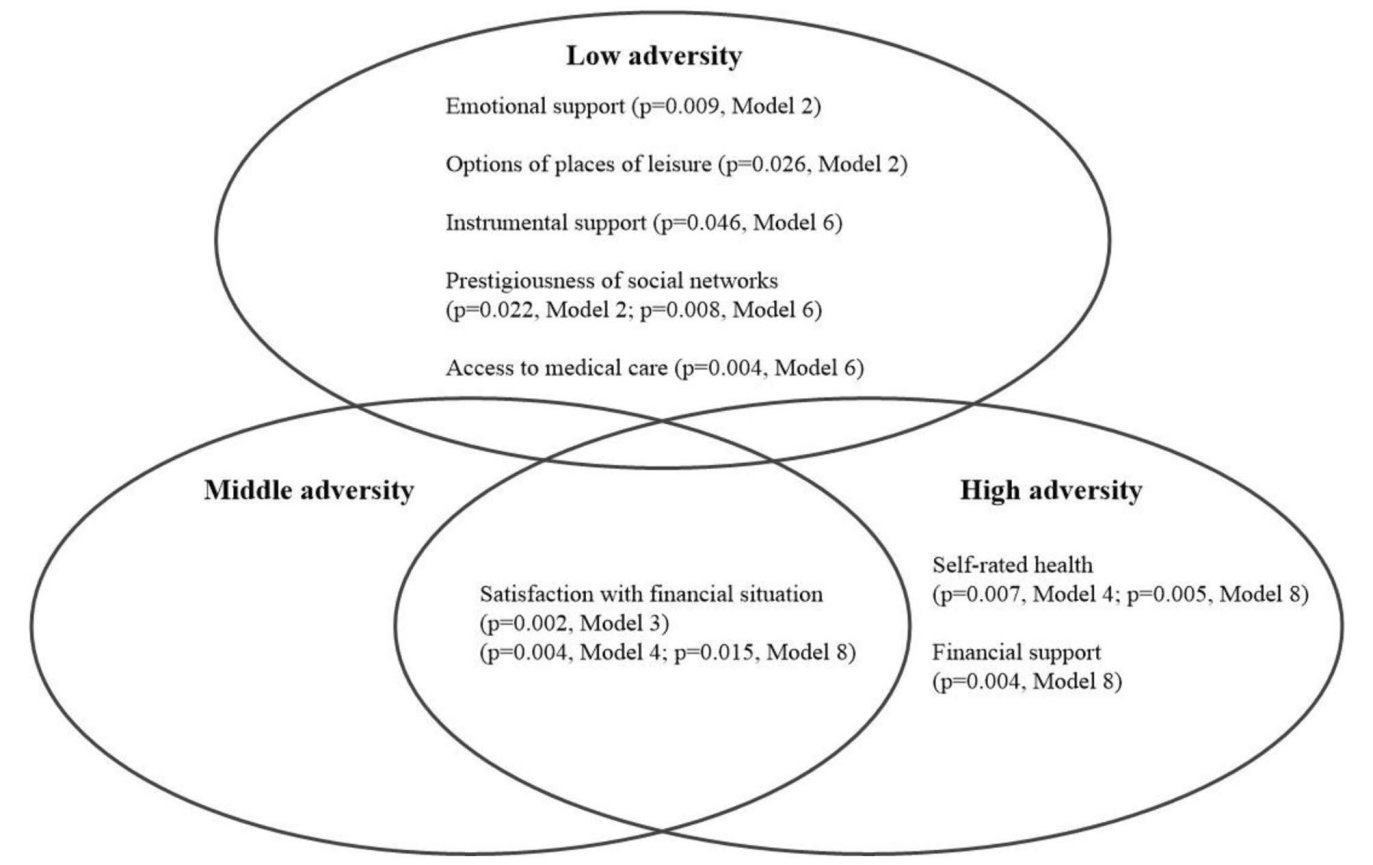
Significant factors of resilience at varying levels of adversity This Venn diagram presents significant factors (*p* < 0.05) that correlate with resilience at different adversity levels among older adults. Each circle is labelled with an adversity level—low, medium, or high—and includes factors significant at that level. The intersecting sections denote factors that are significant across multiple adversity levels Models 2 and 6: Multilevel linear regression of Type-1 and Type-2 resilience, respectively, in the low adversity group Model 3: Multilevel linear regression analysis for predictors of Type-1 resilience in the middle adversity group Models 4 and 8: Multilevel linear regression of Type-1 and Type-2 resilience, respectively, in the high adversity group

Third, the findings of this study suggest that factors related to resilience varied with the level of adversity. For example, resilience among rural older adults in the low-adversity group was mainly promoted by social support (i.e., prestigiousness of the social networks, emotional support, and instrumental support) and environmental factors (i.e., access to medical care and options of places of leisure). Only individuals’ subjective appraisal of their financial situation promoted resilience in the middle-adversity group. Resilience in the high-adversity group was promoted by financial support, which is a social support factor, as well as self-rated health and satisfaction with financial situation, both of which are individual-level resources. The findings also highlight the important role of satisfaction with one’s financial situation, which is the only factor that was associated with resilience in both the middle- and the high-adversity groups (see Fig. 2).

The above result suggests the importance of the subjective attitudes and perspectives of rural older individuals facing multiple challenges, particularly those in the middle- and high-adversity groups. Older adults in rural areas have limited resources that may be further depleted when they encounter diverse adversities—presenting additional challenges to their financial and social resources. Under such situations, a positive attitude can provide a buffer from external stressors even though it may not be grounded in reality, and can help older adults acknowledge their vulnerability, and accept physical or psychological changes in a demonstration of their self-awareness and self-esteem [5, 36, 38, 39, 74].

### Limitations

This study has several limitations. First, the residual approach used to operationalize resilience is based on the assumption of a linear relationship between adversity and life satisfaction/depressive symptoms. However, other possible relationships may exist between these factors, such as those represented by nonlinear threshold models, asymptotic patterns, and inverted U-shaped and challenge models [53]. In addition, given that different stressors may have varying impacts on older adults, their adversity may not necessarily be equivalent to the total number of adverse events that they have experienced. Furthermore, adversity used to operationalize resilience in this study does not include a comprehensive list of all possible adversities experienced by older adults, such as daily hassles, which may have a transient negative emotional impact that dissipates within a day or two. Nevertheless, the repeated and accumulated occurrence of daily events over time can exacerbate emotional distress. It is also noteworthy that our operationalization of resilience focused on residuals of life satisfaction and depressive symptoms, and thus the underlying mechanisms through which these factors influence people’s overall resilience requires further examination. Finally, the data from rural areas of Anhui province may not fully reflect the varied conditions across China’s numerous rural regions. Therefore, extrapolating these findings to other rural areas should be undertaken with a thorough consideration of the regional disparities. Future research should endeavor to compare resilience factors in various rural contexts to foster a more comprehensive understanding of resilience among rural older adults.

## Conclusions

In this study, we found that resilience among older adults in rural China is associated with individual factors, social support, and environmental factors, and those factors play different roles for older adults with different levels of adversity. Especially, we examined two types of resilience focusing on life satisfaction and depressive symptoms and the results showed that older adults may exhibit both types of resilience, only one of them, or neither. While some predictive factors, such as satisfaction with financial situation, consistently influenced both types of resilience, others, like intergenerational support and access to medical care, showed variability. The resilience observed among older adults in rural China is distinctly influenced by traditional values, such as filial piety, which shape their response to adversity. A culturally related finding was the negative correlation between access to geriatric care services and resilience. This insight opens avenues for further research into the cultural dimensions of aging and acceptance of institutional care in rural settings. Therefore, interventions to enhance resilience should be culturally congruent and tailored to individuals’ psychological needs, their levels of adversity, and their receptivity to various forms of support. Ultimately, our study underscores the need for a culturally sensitive approach in both research and practice to effectively understand and support resilience among aging populations in diverse cultural settings.

## Data Availability

The datasets used in this study will not be made publicly available due to the fact that the data involves personal information, but are available from the corresponding author on reasonable request.

## Abbreviations

CHARLS: China Health and Retirement Longitudinal Study
PTSS: post-traumatic stress symptoms
PWB: psychological well-being
ADL: basic activities of daily living
IADL: instrumental activities of daily living
SD: standard deviation
ICC: intraclass correlation coefficient
